# Therapeutic Drug Monitoring of GS-441524 in Cats with Feline Infectious Peritonitis: Pharmacokinetic Variability and Implications for Dose Optimization

**DOI:** 10.3390/pathogens15030291

**Published:** 2026-03-06

**Authors:** Stephen W. Cooke, Rachael Hammond, Danièlle A. Gunn-Moore

**Affiliations:** 1 The Microsampling Laboratory Ltd., Oxfordshire OX10 7EX, UK; 2 Royal (Dick) School of Veterinary Science, The University of Edinburgh, Edinburgh EH25 9RG, UK; rachael.hammond@ed.ac.uk (R.H.); danielle.gunn-moore@ed.ac.uk (D.A.G.-M.)

**Keywords:** GS-441524, remdesivir, feline infectious peritonitis, FIP, treatment, Therapeutic Drug Monitoring, dosage, plasma concentration, cat, HPLC

## Abstract

Remdesivir (REM) and its parent drug, GS-441524 (GS-44), are used to treat cats with feline infectious peritonitis (FIP), resulting in a survival rate of circa 85% (range 77–96%). Cats suffering from FIP exhibit complex and variable clinical presentations, which will cause concurrent variations in the pharmacokinetics (PK) of GS-44. In turn, this will vary the ability of target cells (monocytes and tissue macrophages) to absorb GS-44 in sufficient quantities to achieve optimal antiviral efficacy, resulting in full recovery. Sparse data exists to guide treatment regimens optimized for every presentation of FIP. Therapeutic Drug Monitoring (TDM) measured the GS-44 concentration in 728 blood samples from 263 cats undergoing treatment and generated 173 PK graphs. These identified individuals varied in their ability to absorb GS-44, leading to sub-optimal (11%) and supra-optimal (12%) dose-normalized plasma concentrations. Dosage alterations were suggested to guide subsequent dosages to ensure optimum GS-44 concentrations for individual cats (the clinical outcome report will be published separately). Proposed TDM target values are: area under the concentration vs. time curve (AUC), at least 220 µM·h, time per day that plasma concentration exceeds 3 µM, at least 23 h, time per day plasma concentration exceeds 10 µM, and at least 9 h.

## 1. Introduction

Remdesivir (REM) and GS-441524 (GS-44) are antiviral agents that are safe and effective when used to treat cats suffering from various forms of feline infectious peritonitis (FIP) [1,2]. Both drugs are now legally available to veterinary surgeons in the UK, the US, and many other countries under prescription from licensed veterinary pharmaceutical compounding companies as “Specials”.

The treatment regimens being used for these drugs are based on pharmacokinetic (PK) data derived from studies involving in vitro cell cultures and healthy cats [3,4,5,6,7,8], as well as retrospective reports of results from cats treated with legally sourced GS-44 and/or REM [4,7,8,9,10,11,12,13,14,15,16,17,18,19,20] or unverifiable compounds sourced illegally [21,22,23,24,25]. To date, published PK and pharmacodynamic (PD) data for these drugs being administered under veterinary supervision to cats with FIP are sparse [4,7,8,11], and robust investigations are needed into dosages appropriate for each FIP phenotype and clinical presentation [8].

Therapeutic Drug Monitoring (TDM) is used to monitor the plasma concentration of drugs to optimize treatment [26], preventing sub-optimal treatment (leading to treatment failure and/or drug resistance) and/or overdosage (leading to potential toxicity, other side effects, and/or unnecessary cost). GS-44 has been shown to apparently cure approximately 85% (reported range 77–96%) [3,7,9,12,14,16,19,20] of cats suffering from FIP. However, sub-optimal dosing leads to treatment failure, a partial response requiring longer treatment courses, or a remission state (i.e., the cat no longer shows overt signs of illness, yet the virus remains sequestered in its body) from which relapse may occur at a later date [7,9,11,27].

Of particular concern is that some individuals relapse from remission with neurological and/or ocular signs (which may not have been present at the initial presentation) [9,10,11]. This may indicate sequestration of FIP virus (FIPV) within the central nervous system (CNS) and/or eyes, where it had been protected from adequate concentrations of GS-44 by the blood–brain barrier and/or blood–retina barrier [10,11]. Another concern is overdosage, which may cause the formation of GS-44 calculi in the urinary tract and subsequent bladder and/or kidney pathology [28,29], as well as being more costly.

TDM has historically been associated with the use of a single trough sample value to predict if the associated dose was adequate for the purpose. However, this approach may not always be appropriate when giving potentially toxic drugs [26] or in cats suffering from FIP, where there is no significant correlation between trough concentrations and clinical efficacy [8].

The aims of this study were (1) to determine plasma GS-44 concentrations in cats suffering from the various phenotypes of FIP being treated with GS-44 and REM, (2) develop a range of PK/PD index values for the study population, (3) use paired TDM sampling and identify cats exhibiting PK/PD indices that vary from those exhibited by the majority of the study population, and (4) determine if, for cats exhibiting abnormal indices, dosage modifications could correct that variation.

## 2. Materials and Methods

### 2.1. Patient Recruitment and Sample Collection

The blood samples collected in this study are from cats receiving UK legally sourced GS-44 or REM (from BOVA Veterinary Specials or Summit Veterinary Pharmaceuticals) prescribed by their veterinary surgeon for the treatment of FIP diagnosed as “confirmed” or “very likely”, as per ABCD FIP Guidelines [1]. The study was approved by the Veterinary Ethical Review Committee (VERC) of the University of Edinburgh, UK (VERC 63.22; dated 4 December 2022).

Under the care and control of their attending clinicians and with informed owner consent, blood samples were collected from these cats and sent to the Easter Bush Pathology laboratory of the Royal (Dick) School of Veterinary Studies (RDSVS), The University of Edinburgh, for routine analysis, e.g., hematology, biochemistry, and/or acid 1 alpha glycoprotein (AGP) assay. Residual serum or plasma samples were sent to The Microsampling Laboratory Ltd. (MSL) for assay and GS-44 concentration. The cat’s weight, the dosage and presentation of GS-44 dispensed, the time the last dose was administered, and the time the blood sample was collected were recorded.

Samples were stored at fridge temperature (46 °C) and transported at ambient temperature. Analysis was performed within 48 h of sample receipt at MSL.

The study cats provided a broad range of patient presentations (i.e., age, sex, neuter status, breed, weight, husbandry, FIP phenotype [non-effusive, effusive, neurological, ophthalmic, or a mixture of those phenotypes], duration and stage of illness, complicating pathologies, concomitant treatments, dosage and mode of administration of antiviral drugs, time under treatment, and client compliance).

### 2.2. Healthy Cat GS-44 PK Graphs

**Figure 1:**
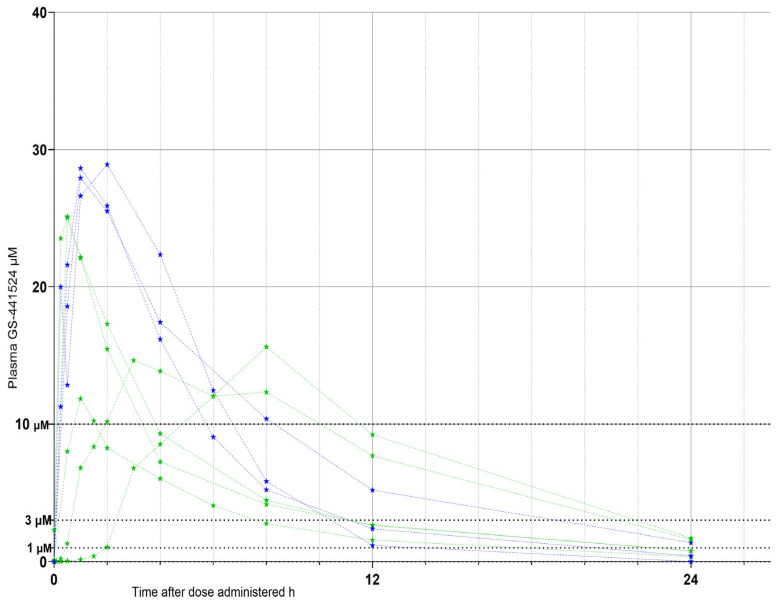
The plasma concentration of GS-441524 (GS-44) in healthy cats administered either remdesivir (REM) or GS-44 at a (normalized) dose of 20 mg/kg, at time = 0, with sequential blood samples assayed for GS-44 (µM) and plotted as concentration vs. time. The drug was administered subcutaneously (blue dotted lines & stars) or orally (green dotted lines & stars). Breakpoint concentrations of 1 µM, 3 µM, and 10 µM were added to provide an indication of the accepted effective concentration values (rounded up to whole numbers) (black horizontal dotted lines) for the inhibition of FIPV RNA replication in vitro and in vivo. Data extracted by digitization from published data [3,4,5,6].

Healthy cat GS-44 PK graphs were derived from published reports, which included graphs depicting plasma concentration of GS-44 vs. time [3,4,5,6]. The concentration and time values were derived by digitizing (PlotDigitizer, v. 3.3.7, 2024) these graphs. Twelve graphs describing the concentration/time relationships for subcutaneous (sc), oral (po), and intravenous (iv) administration of GS-44 were obtained. Since our study included only two samples from cats receiving iv REM, the four healthy cat iv datasets were excluded. The data for the remaining eight graphs were used in this study (Figure 1). As the various studies used different doses of REM and GS-44, the results were normalized to a 20 mg/kg dose, a value chosen to both visually fit the range of study sample GS-44 concentrations and which was representative of the current (July 2025) recommended doses used for the various FIP phenotypes (10–20 mg/kg) [1,2].

When REM is administered parenterally, it is rapidly converted to GS-44 [30] in an equimolar ratio, so REM doses were converted to an equivalent dose of GS-44 using its salt factor 0.483 (molecular weight GS-44 {291.26} ÷ molecular weight REM {602.60}).

Breakpoint concentrations of 1 µM [3], 3 µM [6], and 10 µM [3] were added to provide Figure 1 with an indication of the accepted effective concentration (EC) values (rounded up to whole numbers) for the inhibition of FIPV RNA replication in vitro and in vivo [3,6].

The doses used were clinically varied, so each study sample result was normalized; hence, normalization calculations using 10 mg/kg/dose, 15 mg/kg/dose, and 20 mg/kg/dose were performed. The visual fit of the 10 mg/kg/dose and 20 mg/kg/dose values to the study data was not as close as for the 15 mg/kg values (Supplementary Data Figures S1 and S2); hence, the 15 mg/kg per dose ([assay raw result ÷ dose as administered] × 15), designated “N15”, is presented within this paper. First-order elimination of GS-44 was assumed [31], where the concentration is proportional to the dose; hence, increasing the dose proportionally increases the assay result value.

The healthy cat normalization value of 20 mg/kg was empirically equivalent to a study cat normalization value of 15 mg/kg; the difference was presumed to be due to the effects of individual cat variability, FIP pathology, and/or variations within experimental methodology used.

### 2.3. Study Cat Result Definitions

**Figure 2:**
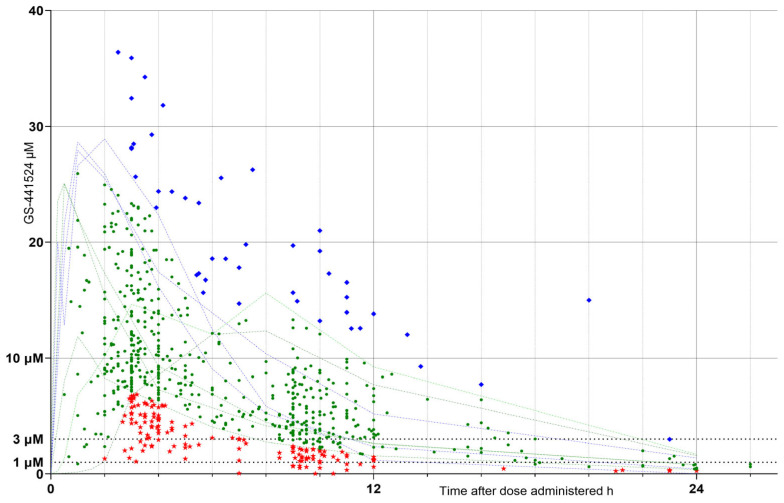
Sample GS-441524 concentration (µM) vs. time (h) after each dose administered. Sample values (“raw” results) falling outside and below the healthy cat response ranges = low raw response cohort (LOW raw RC), red stars; those falling within the healthy cat response ranges = optimum raw (OPT raw RC), green circles and those falling outside and above the healthy cat response ranges = high raw (HIGH raw RC), blue diamonds. Faint green and blue dotted lines indicate data obtained from healthy cat responses, extracted by digitization of published data [3,4,5,6] for comparison (as in Figure 1) and scaled to a 20 mg/kg dose (see Figure 1). Markers at 1, 3, and 10 µM indicate concentrations bracketing effective viral inhibition values [3,6], as in Figure 1.

**Figure 3:**
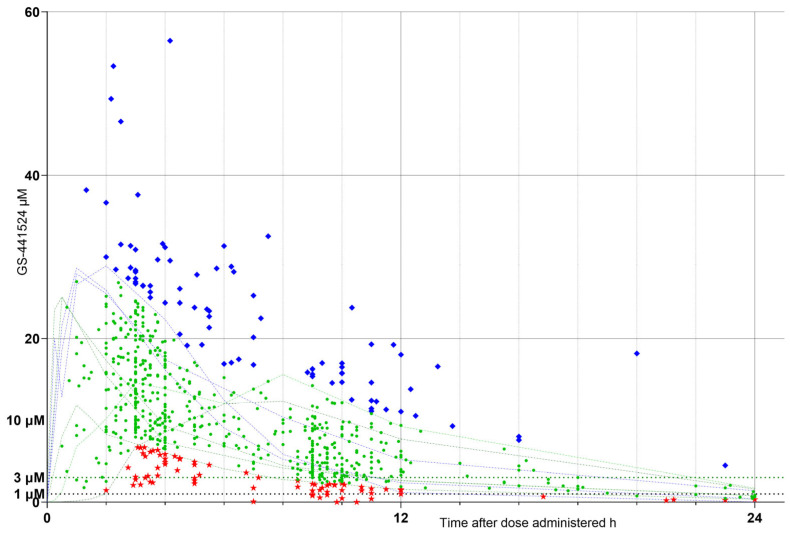
Sample GS-441524 concentration (µM) vs. time (h) after dose administered; assay values normalized to 15 mg/kg per dose (“N15”) to remove dosage variability. Sample values falling outside and below the healthy cat response ranges = low N15 response cohort (LOW N15 RC), red stars; those falling within the healthy cat response ranges = optimum N15 (OPT N15 RC), green circles; and those falling outside and above the healthy cat response ranges = high N15 (HIGH N15 RC), blue diamonds. Faint green and blue dotted lines indicate data obtained from healthy cat responses, extracted by digitization of published data [3,4,5,6] for comparison (as in Figure 1) and scaled to a 20 mg/kg dose (see Figure 1). Markers at 1, 3, and 10 µM indicate concentrations bracketing effective viral inhibition values [3,6] as in Figure 1.

The raw results were plotted as concentration (µM) versus (v) time after dose administration (h) (Figure 2). Based on its relationship to the range of healthy cat PK graphs (faint green and blue lines on Figure 2 and Figure 3), each result was assigned to a response cohort (RC) defined as:

(1) **Low response (LOW raw RC),** being samples with values falling outside and below the healthy cat response ranges (red stars on the graphs);
(2) **Optimum response (OPT raw RC),** those falling within the healthy cat ranges (green circles);
(3) **High response (HIGH raw RC),** those falling outside and above the healthy cat ranges (blue diamonds).

The normalized, N15, results were similarly plotted (Figure 3) and, using the same RC definitions, populated N15 RCs (LOW N15 RC, OPT N15 RC, and HIGH N15 RC).

### 2.4. TDM and PK Sampling

Paired TDM samples, consisting of a peak sample and taken close to the expected time at which the maximum concentration (Cmax) occurred (nominally at dose +3 to 5 h), and a trough sample, taken closer to the time of the next dose (nominally at dose +9 to 11 h), were preferred. Single samples were also accepted as long as the time after dosing was recorded.

Where paired TDM samples from one cat were obtained, graphs of GS-44 concentration vs. time over a 24 h period were constructed from the results using the Sawchuk–Zaske steady-state method, with the area under the sample drug concentration vs. time curve (AUC) being calculated using the linear-up/log-down method [32,33], with the time that Cmax would likely occur (tmax) being set at 2.0 h, a value chosen by considering the healthy cat data [3,4,5,6]. A drug steady state was assumed as all study cats had been administered a constant dose of GS-44 for at least 3 days prior to sampling. These graphs represented the study cat equivalent of the data shown in Figure 1.

The PK indices of Cmax, half-life (t1/2), and true trough (the concentration at the time of the next dose [Tt]) were calculated using the dose per dose (mg/kg/dose).

The AUC for a 24 h period (AUC0–24) and the times at which the sample concentration exceeded the target concentrations of 3 µM and 10 µM over a 24 h period (t > 3 µM and t > 10 µM) were calculated using the dose given over 24 h (mg/kg/day).

Given the limited GS-44 pharmacokinetic data available for cats with the various FIP phenotypes, and recognizing that trough-only TDM, where a single sample is collected immediately before dosing, offers minimal insight into the pharmacokinetic behaviour of agents such as GS-44 [8], we based our TDM pharmacokinetic assessments on paired samples. Consequently, we did not attempt to construct population pharmacokinetic models for this study.

### 2.5. Dose–Response Cohort (DRC) Definition

Raw RC values and their corresponding normalized 15 mg/kg/dose (N15 RC) designations were compared for each sample, allowing assignment to one of five DRCs:

Low absorbing (LOA DRC): Samples with LOW raw RC values that, after normalization to a 15 mg/kg/dose, remained below the lower limit of the healthy cat reference intervals (RIs). These results are hypothesized to originate from cats with reduced oral absorption of GS-44; thus, even escalation to 15 mg/kg may not achieve plasma concentrations expected in healthy cats.

Low dose (LOD DRC): Samples with LOW raw RC values that, when normalized to a 15 mg/kg/dose, fell within the healthy cat reference range. These results are hypothesized to reflect cats that received doses lower than those given to most study or healthy cats. Increasing the administered dose to 15 mg/kg would be expected to produce plasma concentrations comparable to those of healthy cats.

High dose (HIGH DRC): Samples with HIGH raw RC values that, after normalization to a 15 mg/kg/dose, fell within the healthy cat reference range. These results are hypothesized to reflect cats that received doses higher than those typically administered. Reducing the dose to 15 mg/kg would be expected to yield plasma concentrations within the healthy cat reference range.

High absorbing (HIA DRC): Samples with HIGH raw RC values that, even after normalization to a 15 mg/kg/dose, remained above the upper limit of the healthy cat reference range. These results are hypothesized to represent cats with enhanced oral absorption of GS-44 such that, even at 15 mg/kg, plasma concentrations would be expected to exceed those of healthy cats.

Optimal (OPT DRC): Samples with OPT raw RC values that remained within the healthy cat reference range after normalization to a 15 mg/kg/dose. These samples are hypothesized to originate from cats whose liberation, absorption, distribution, metabolism, and elimination (LADME) characteristics are broadly comparable to those of healthy cats.

TDM pairs were assigned to a DRC according to the DRC classification of the constituent samples. Pairs in which both samples were classified as OPT DRC were designated as OPT DRC; if one or both samples were classified outside the OPT DRC, the pair was assigned to the corresponding non-OPT DRC.

For each PK index (Cmax, t_1/2_, Tt, AUC_0–24_, t > 3 µM, and t > 10 µM), the five DRC cohorts were analyzed using GraphPad’s embedded statistical procedures (ordinary one-way ANOVAR with Tukey’s multiple comparison test).

Data were plotted only when a statistically significant difference between the group means was identified (*p* < 0.05). Pharmacokinetic indices derived from the Sawchuk–Zaske method, volume of distribution (Vd), clearance at steady state (CLss), and the elimination rate constant (kel) are presented in the Supplementary Materials for reference (Tables S1–S3).

### 2.6. Bioanalytical Method

All TDM assays were performed at MSL by a single analyst (author S.W.C.) using a high-performance liquid chromatography (HPLC) with a diode array detection method developed and validated at MSL. Briefly, aliquots of serum or plasma were protein precipitated with 100% methanol at 1:5.5 and 1:10 dilutions. After centrifugation, the methanol was removed by vacuum and replaced with an equal volume of HPLC mobile phase (96% water, 4% acetonitrile acidified with 0.1% 100% formic acid) and separated, using a Waters XSelect Premier HSS T3 100A, 2.5 µm, 4.6 × 150 mm column, by isocratic HPLC. GS-44 was detected at 240 +/− 10 nm and quantified using external calibration (between 16 and 5048 ng/mL, equivalent to sample concentrations of between 0.30 and 173.0 µM). GS-44 concentrations were reported as the average of the two assay results.

### 2.7. Statistical Analysis

Statistical analyses were performed using Microsoft Excel (Microsoft, Redmond, WA, USA) and GraphPad Prism for Windows v10.2.3 (GraphPad Software, Boston, MA, USA). Non-parametric comparisons among the five DRC cohorts were conducted using ordinary one-way ANOVAR with Tukey’s multiple comparison test. A *p*-value of <0.05 was considered statistically significant.

## 3. Results

A total of 728 clinical blood samples were collected from 263 cats. The “raw” sample GS-44 concentrations (µM) vs. time in hours (h) after dose administered were plotted (Figure 2). Also plotted as comparative markers are the healthy cat PK graphs (faint green and blue lines) and *y*-axis markers at 1, 3, and 10 µM.

In Figure 2, LOW raw RC are shown as red stars, OPT raw RC as green circles, and HIGH raw RC as blue diamonds. Faint green and blue dotted lines indicate data obtained from “healthy cats”, extracted by digitization of published data [3,6] and scaled to a 20 mg/kg dose (see Figure 1 and Figure 2). Markers at 1, 3, and 10 µM indicate concentrations bracketing effective viral inhibition values [3,6].

### 3.1. Dosages

Clinical doses (Table 1) ranged between 2.76 and 28.0 mg/kg (mean 11.98 mg/kg), which included doses not currently recommended [2].

The “raw” sample GS-44 concentrations (µM) vs. time in hours (h) after dose administered, shown in Figure 2, were dose-normalized to a 15 mg/kg/dose, and the values were plotted (Figure 3). Also plotted as comparative markers are the healthy cat PK graphs (faint green and blue lines) and y-axis markers at 1, 3, and 10 µM.

In Figure 3, LOW N15 RC are shown as red stars, OPT N15 RC as green circles, and HIGH N15 RC as blue diamonds.

The numerical distribution of the study sample RCs derived for both raw and N15 values is shown in Table 2

The numerical distribution of the study sample DRCs, as determined from the RC results, is shown in Table 3.

### 3.2. Pharmacokinetic (PK) Indices

From the study samples, 173 usable TDM pairs were obtained. The peak and trough samples were (ideally) obtained on the same day (140 pairs) or less than 14 days from each other (33 pairs). TDM pairs were collected from 106 individual cats, and of these, 67 cats produced single pairs, 48 cats produced two pairs, eight cats produced three pairs, five cats produced four pairs, one cat produced six pairs, and one cat produced eight pairs.

### 3.3. Cmax at 2 h Post-Drug Administration

**Figure 4:**
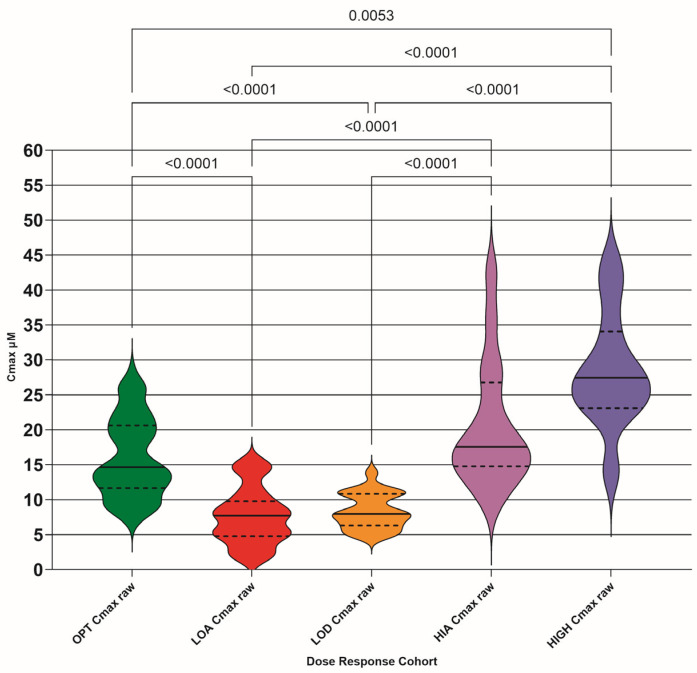
Calculated maximum concentration (Cmax) of GS-441524 for each TDM pair (µM) for each dose–response cohort (DRC); optimum (OPT AUC raw), low absorber (LOA AUC raw), low dose (LOD AUC raw), high absorber (HIA AUC raw), and high dose (HIGH AUC raw). Differences between the means are only shown if they are significant (<0.05). Median (solid lines) with 95% confidence interval of difference (interrupted lines) is also added.

**Figure 5:**
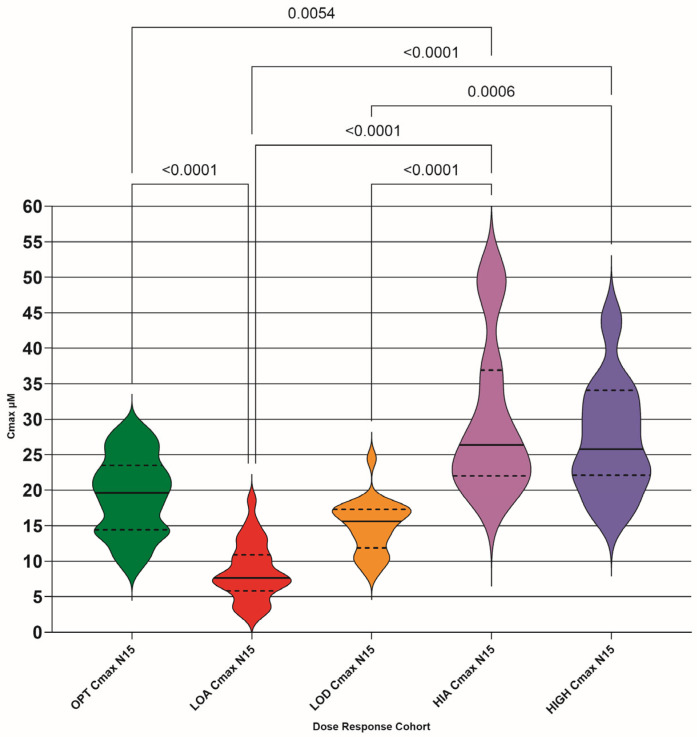
Calculated maximum concentration (Cmax) of GS-441524 for each TDM pair (µM) normalized to 15 mg/kg (N15) for each dose–response cohort (DRC); optimum (OPT Cmax N15), low absorber (LOA Cmax N15), low dose (LOD Cmax N15), high absorber (HIA Cmax N15), and high dose (HIGH Cmax N15). Differences between DRC means are only shown if they are significant (<0.05). Median (solid lines) with 95% confidence interval of difference (interrupted lines) is also added.

The calculated Cmax (µM) for each TDM pair was plotted as violin plots for each DRC (raw values shown in Figure 4 and N15 values shown in Figure 5). The numerical distribution of both raw and N15 values is shown in Table 4.

### 3.4. Half-Life (t1/2)

**Figure 6:**
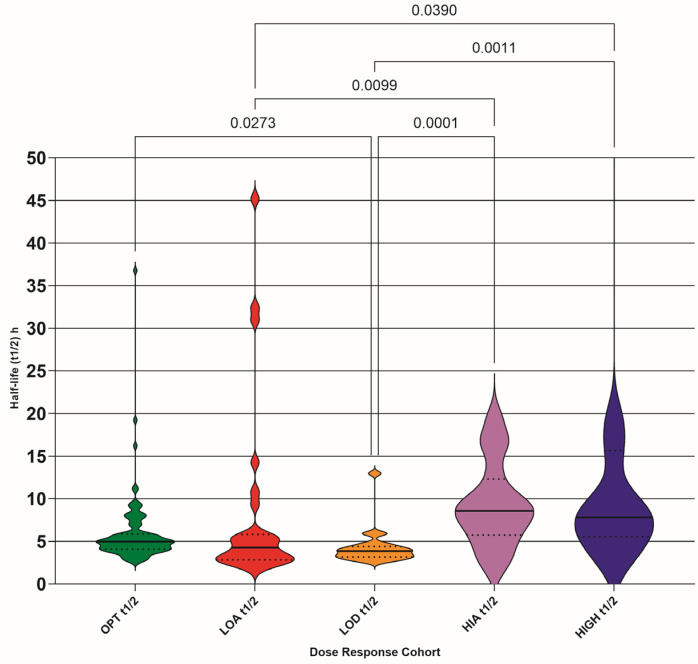
Calculated half-life (t1/2) of GS-441524 for steady-state dosing (h) for each dose–response cohort (DRC); optimum (OPT t1/2), low absorber (LOA t1/2), low dose (LOD t1/2), high absorber (HIA t1/2), and high dose (HIGH t1/2). Differences between DRC means are only shown if they are significant (<0.05). Median (solid lines) with 95% confidence interval of difference (interrupted lines) is also added.

The calculated t1/2 (h) for each TDM pair was plotted as violin plots for each DRC (raw values shown in Figure 6). The numerical distribution of the values is shown in Table 5.

### 3.5. True Trough (Calculated Plasma Concentration Immediately Preceding the Next Dose (Tt)

**Figure 7:**
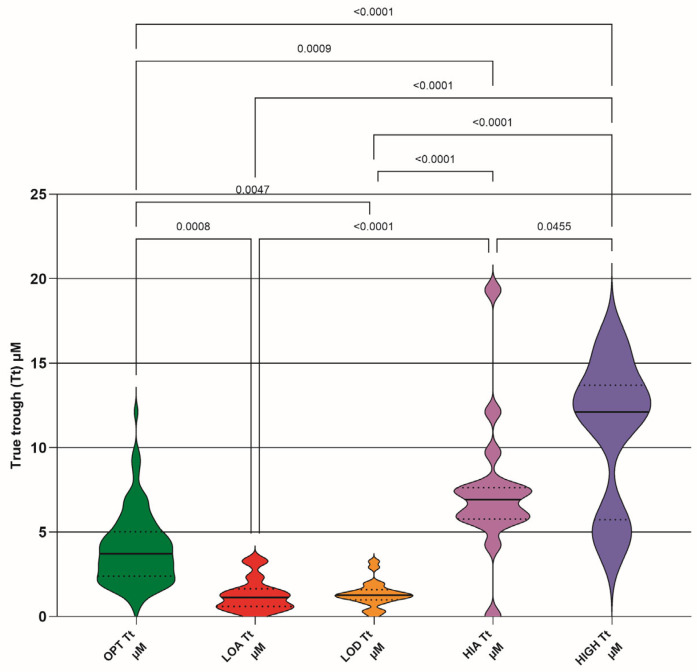
Calculated true trough (Tt) of GS-441524 for steady-state dosing (µM) for each dose–response cohort (DRC); optimum (OPT Tt), low absorber (LOA Tt), low dose (LOD Tt), high absorber (HIA Tt), and high dose (HIGH Tt). Differences between DRC means are only shown if they are significant (<0.05). Median (solid lines) with 95% confidence interval of difference (interrupted lines) is also added.

Calculated true trough (Tt) of GS-44 for steady-state dosing (µM) for each DRC (Figure 7, Table 6); optimum (OPT Tt), low absorber (LOA Tt), low dose (LOD Tt), high absorber (HIA Tt), and high dose (HIGH Tt). Differences between DRC means are only shown if significant (<0.05).

**Figure 8:**
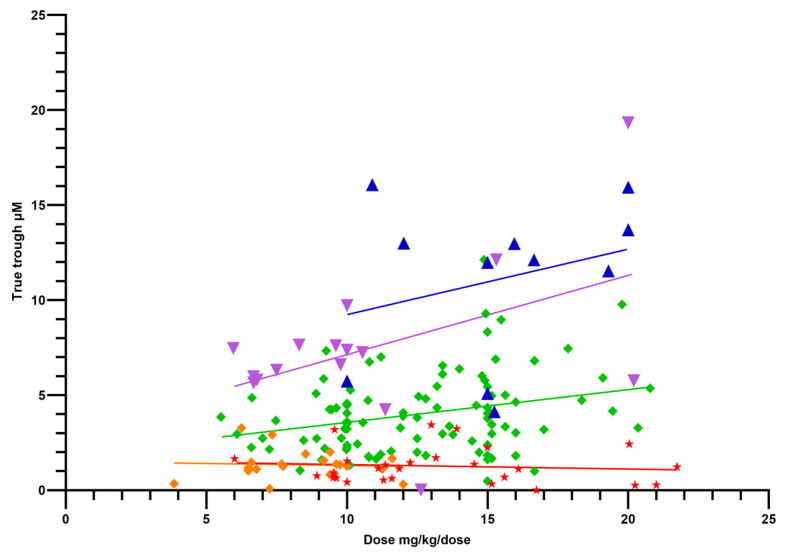
Dose of GS-441524 administered per dose (mg/kg/dose) and the corresponding true trough concentration (µM) for each dose–response cohort (DRC). Green diamond = OPT DRC, red star = LOA DRC, orange diamond = LOD DRC, purple triangle = HIA DRC, blue triangle = HIGH DRC. Lines indicate respective (by color) simple linear regression for each DRC.

To test if Tt values could be used as predictors of PK parameters, the doses administered (mg/kg/dose) were plotted against Tt values (Figure 8) for each DRC. Simple regression analysis is also plotted. Only the regression slope of the OPT DRC (green line) deviated significantly (*p* = 0.006) from zero, suggesting that Tt values did not provide useful information regarding the LADME of GS-44 in FIP cats.

### 3.6. Area Under the Concentration vs. Time Curve for 24 h (AUC0–24)

**Figure 9:**
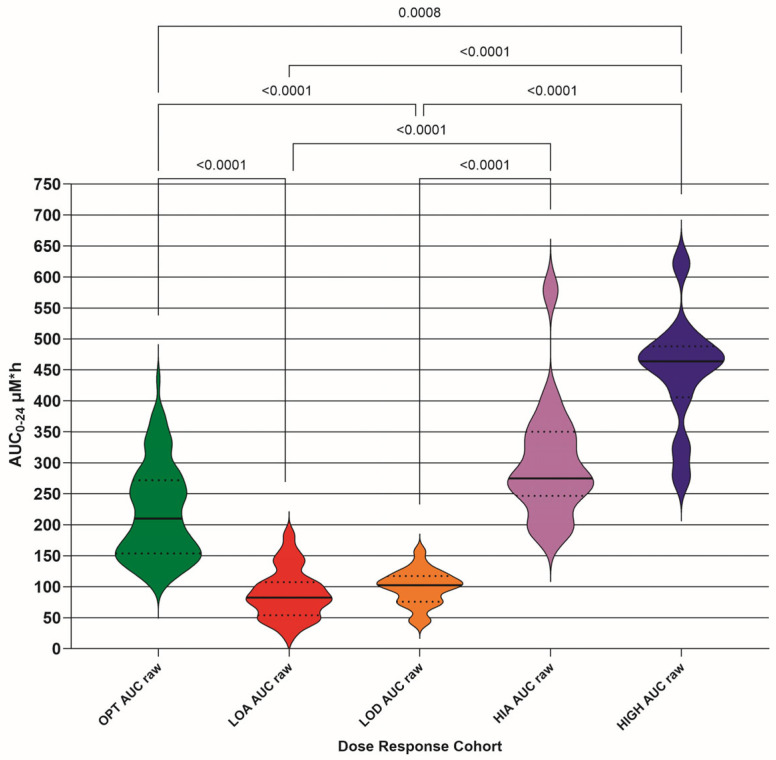
Calculated area under the concentration vs. time curve for 24 h (AUC0–24) (µM·h) for each dose–response cohort (DRC) raw concentration; optimum DRC (OPT AUC raw), low absorber DRC (LOA AUC raw), low dose DRC (LOD AUC raw), high absorber DRC (HIA AUC raw), and high dose DRC (HIGH AUC raw). The differences between means for each DRC are only shown if they are significant (<0.05). Median (solid line) with 95% confidence interval of difference (fine interrupted lines) is also added.

**Figure 10:**
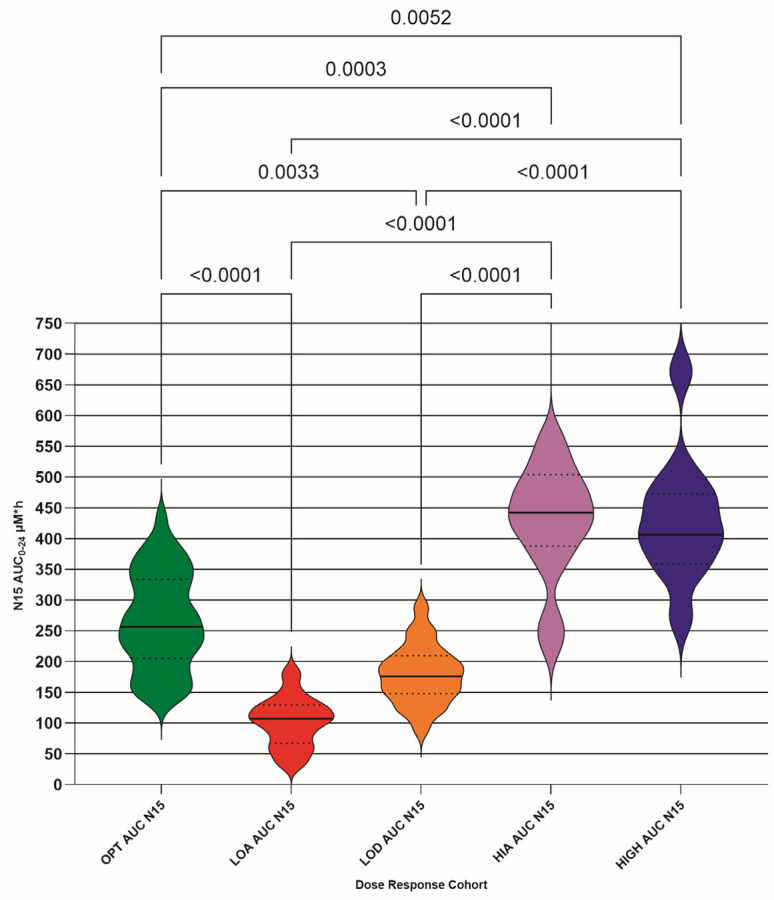
Calculated area under the concentration vs. time curve for 24 h (AUC0–24) (µM·h) for each dose–response cohort (DRC) normalized to 15 mg/kg/day (N15); optimum DRC (OPT AUC N15), low absorber DRC (LOA AUC N15), low dose DRC (LOD AUC N15), high absorber DRC (HIA AUC N15), and high dose DRC (HIGH AUC N15). The differences between means for each DRC are only shown if they are significant (<0.05). Median (solid line) with 95% confidence interval of difference (fine interrupted lines) is also added.

The AUC0–24 was calculated (µM·h) for each DRC; both raw (Figure 9, Table 7) and N15 (Figure 10, Table 7) concentrations were plotted. The optimum (OPT AUC raw/N15), low absorber (LOA AUC raw/N15), low dose (LOD AUC raw/N15), high absorber (HIA AUC raw/N15), and high dose (HIGH AUC raw/N15) are included. Differences between means for each DRC are only shown if they are significant (<0.05).

### 3.7. Time That the Concentration Exceeded Time (h) Target Values

**Figure 11:**
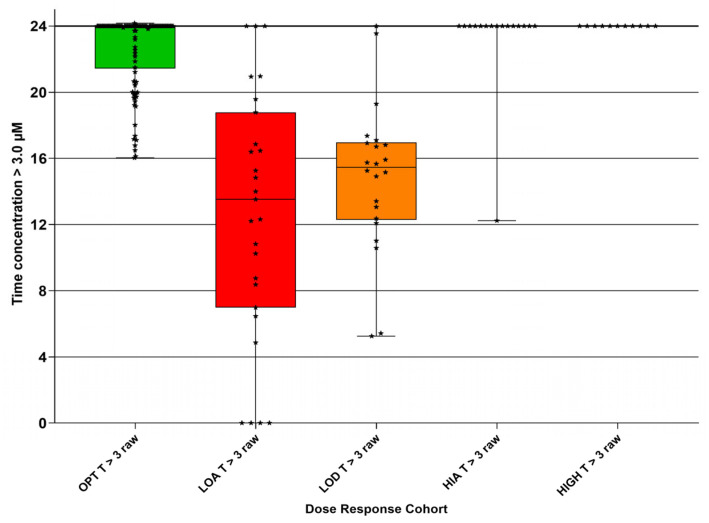
Calculated duration (h) that concentration of GS-441524 exceeded 3 µM in each 24 h period for each dose–response cohort (DRC); optimum DRC (OPT T > 3 raw), low absorber DRC (LOA T > 3 raw), low dose DRC (LOD T > 3 raw), high absorber DRC (HIA T > 3 raw), and high dose DRC (HIGH T > 3 raw). Median (solid line) with 95% confidence interval of difference (extent of colored bars) with results (stars).

**Figure 12:**
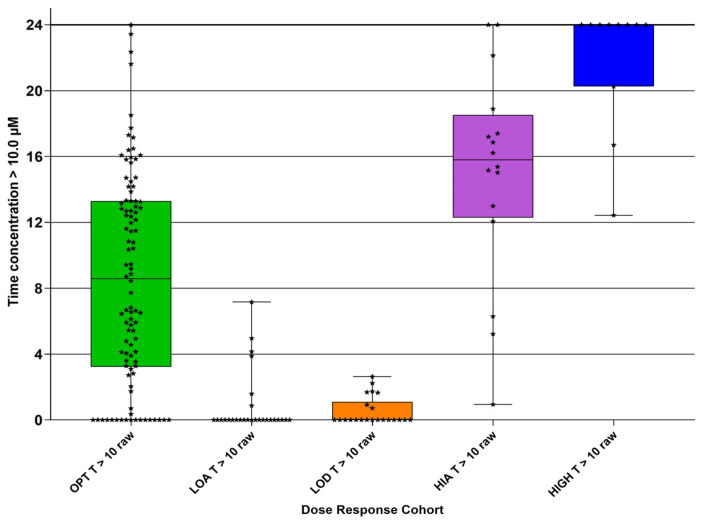
Calculated duration (h) that concentration of GS-441524 exceeded 10 µM in each 24 h period for each dose–response cohort (DRC); optimum DRC (OPT T > 10 raw), low absorber DRC (LOA T > 10 raw), low dose DRC (LOD T > 10 raw), high absorber DRC (HIA T > 10 raw), and high dose DRC (HIGH T > 10 raw). Median (solid line) with 95% confidence interval of difference (extent of colored bars) with results (stars).

The duration (h) within a 24 h dosing interval during which plasma concentrations exceeded 3 µM (T > 3) (Figure 11 and Table 8) and exceeded 10 µM (T > 10) is shown (Figure 12 and Table 8).

## 4. Discussion

The overall mortality rate among cats with FIP treated with the antiviral agents REM and GS-44 is estimated at approximately 15% [2]. Using recent estimates of the global companion cat population (3.50–3.73 × 10^8^) [34] and an FIP incidence of 0.3–1.4% [1], this corresponds to a potential maximum of approximately 780,000 deaths annually. This figure represents a substantial disease burden, which is likely underestimated when feral cats and other Felidae are considered. A striking illustration of the potential impact of FIP occurred in 2023, when a novel recombinant FIPV strain resulted in the deaths of several thousand cats on the island of Cyprus within just a few months [35].

It is incumbent on veterinary professionals to endeavor to reduce FIP-associated mortality, especially if it is demonstrated that the approximately 15% figure does not represent an invariable, inevitable factor in the pathogenesis of FIP.

Early diagnosis and intensive early treatment have been shown to result in a mortality rate nearer 5% [18]. That study also reported the same mortality rate for a shorter, 42-day, treatment period compared to an 84-day treatment period. This indicates that the 15% mortality rate has a realistic potential to be reduced, and our study suggests that other factors affecting the mortality rate can be identified and mitigated using TDM.

Our results indicate that there is a wide range of plasma GS-44 concentrations resulting from oral GS-44 being prescribed by veterinary surgeons and that the variability does not disappear with a dose normalization calculation (Figure 2 and Figure 3), indicating that it is not solely caused by variations in dosage. This finding alone suggests that TDM is likely to be beneficial for case management by identifying individuals who may benefit from a dosage tailored to their unique metabolisms.

In some cases, the doses used are lower than those presently recommended, perhaps indicating that out-of-date sources were used for dosage selection. This is understandable, as GS-44 has only been used clinically for a small number of years, and dosage updates have been common, most recently detailed in July 2025 [35]. Here, TDM can supply data to inform clinicians about the dosage required for the individual cats they are treating, rather than relying on more generalized (and changeable) “one-size-fits-all” doses.

It has been reported that lower doses of GS-44 are associated with sub-optimal treatment outcomes, such as subsequent dose increases being required because of inadequate clinical response, incomplete recovery, or relapse from remission [3,7,8,9,14,16,18,20]. However, as other cats in these studies were apparently cured (rather than being in remission) at comparable doses, individual variability in drug LADME may account for these differences in therapeutic response. Insufficient systemic exposure, whether due to poor absorption or enhanced clearance, may, therefore, contribute to treatment failure in some cases, and TDM can be used to identify those individuals.

This variability in systemic exposure is particularly relevant when considering FIP cases in which drug penetration into protected compartments, such as the central nervous system and/or eyes, is required. Cats presenting with neurological and ophthalmic pathotypes of FIP are typically treated with “higher” doses of GS-44 to maximize the likelihood of achieving therapeutic concentrations within the cerebrospinal fluid (CSF) and aqueous humor. This approach is necessitated by the restrictive nature of the blood–brain and blood–retina barriers, which limit drug penetration into these compartments [3]. To date, however, GS-44 concentrations in the CSF or aqueous humor of treated cats have not been reported, and such assumptions remain inferential and unquantified.

Within the LOW raw RC TDM, five samples originated from cats being administered low doses of GS-44 due to dispensing errors (retrospectively identified as being due to faulty compounding of the oral liquid) or owner non-compliance with dosing instructions, both of which were corrected. The TDM identified issues that might otherwise have been misinterpreted, perhaps as viral resistance or an inherent inability to respond to treatment.

At present, GS-44 remains unlicensed, so there are no formal recommendations for dosages derived from robust experimental data analysis (as occurs for licensed drugs). Instead, the doses were suggested by initial studies calculating viral effective concentration (EC) values [10,11]; these were subsequently increased as clinicians found higher dosages were needed [1].

The concentration of a drug that inhibits viral RNA replication by 50% (compared to untreated cells) is EC50; similarly, EC90 causes a 90% inhibition of viral replication. The use of EC values can be used to inform dosage; however, these metrics are best suited to comparing different drug efficacies in the developmental laboratory setting rather than in clinical cases. Healthy laboratory cats are likely to have GS-44 LADME that is dissimilar to the majority of the ill study cats, which may also vary widely by breed, age, husbandry, FIP phenotype, time after FIPV infection, etc. For FIPV, EC values have been determined in vitro using cell types and modified virus lines [3,5,6], which may not be fully representative of the pathological characteristics of naturally occurring FIP. For example, the laboratory studies involved mostly non-pedigree cats [3,5,6], while most natural cases of FIP occur in pedigree cats, typically British Short Hair, Ragdoll, and Maine coon breeds [9]. In addition, treatments were instigated very soon after virus challenge in the laboratory studies, a situation not often achieved in field cases, which will influence treatment outcomes [17]; for example, terminal FIP is associated with systemic suppression of CD4^+^ and CD8^+^ T-lymphocytes necessary for mounting cell-mediated immunity, so these cats are likely to find it much harder than healthy laboratory cats to fight off FIPV infection [36,37].

In order to indicate relevant breakpoints for effective GS-44 concentrations, we have rounded up the reported EC90 continuous extra-cellular concentration values of GS-44 for FIPV [3,6] to 1 µM and 3 µM. Values less than 1 µM are unlikely to provide significant inhibition of viral replication. Between 1 and 3 µM may represent a gray area within which any variation in LADME or viral resistance may result in sub-optimal clinical effect; however, our suggested breakpoint of 10 µM is derived from a clinical FIP case and infected monocytes [3], so it is clinically relevant and allows us confidence that the 1 and 3 µM values are valid comparators. Note that even the 10 µM concentration did not completely inhibit FIPV replication, reducing it by 1000-fold after 20 h and “significantly” after 72 h continuous incubation at this concentration [3].

Inhibiting viral replication either completely or at a level that allows the inherent immune response to remove infected cells effectively is a valid therapeutic goal, and presenting GS-44 to the intended target (monocytes and tissue macrophages infected with FIPV) by means of maintaining a known effective plasma concentration is a valid premise.

The antimicrobial effect of different anti-bacterial drugs may be described as concentration-dependent, time-dependent, or a combination of both, which are defined by three PK indices [38]: (a) minimum inhibitory concentration (MIC); i.e., the lowest concentration of an antimicrobial drug that prevents demonstrable reproduction of a microorganism under the experimental conditions applied; (b) AUC0–24; and (c) Cmax. The ratios AUC0–24/MIC and Cmax/MIC define concentration-dependent antibiotic dose requirements; the time the plasma concentration exceeds the MIC (t > MIC) defines time-dependent antibiotic dose requirements, and AUC0–24 best defines the combination of both. These are the PK/PD indices, and for each drug/organism combination, a pharmacodynamic target (PDT) is established. These parameters are also used to describe the efficacy of antiviral drugs in vitro, with EC values substituting for MIC. However, EC50 or EC90 are not equivalent to a bacterial MIC, so an EC100 value should be used if an equivalent to MIC is sought.

In vivo antiviral PDT values for GS-44 and REM have not been documented. Although TDM was used to optimize treatment of COVID-19 in humans [39,40], it is not known whether GS-44 is a time- and/or concentration-dependent drug. Our data will act as a starting point for determining a relationship for FIPV in cats.

Our study Cmax values show a significant difference between the OPT and LOA, and LOD and HIGH cohorts, suggesting that drug absorption varies in efficiency. The OPT and LOD cohort difference disappeared with normalization, as expected, as the LOD cohort is hypothesized to be caused by lower doses than presently (July 2025) recommended. The OPT and HIA difference appeared with normalization, as expected, as the HIA cohort is hypothesized as being more efficient at drug absorption, so normalizing the dose would increase the amount of drug in the plasma.

The t1/2 values showed less variation between DRCs; however, the range of values was greater and may suggest that elimination (which mainly occurs via the kidneys for GS-44 [8]) could be affected by renal pathology secondary to FIP. This requires further investigation.

The lack of a relationship between dose and Tt (excepting that for the OPT DRC) highlights the fact that a steady state in many cats is independent of dosage given and supports the use of TDM to quantify (in a clinical setting) the actual dose required to produce a known plasma concentration.

The raw AUC0–24 values also show significant differences between OPT and LOA, and LOD and HIGH DRCs. The OPT and HIA cohort difference appeared when the dose was normalized, again as expected. It was not expected that the OPT and LOD difference would remain when the dose was normalized, so it may be that some of the LOD cohort should have been classified as LOA. We deem this not to be an issue for the outcome of the study, as low plasma GS-44 concentrations would be revealed by TDM where they might otherwise not have been.

The T > 3 µM and T > 10 µM results are presented using bar plots (and not violin plots), as the latter do not suit data with a high cut-off value (here 24 h). Data points are also included to allow a meaningful visualization of the study results.

Despite the fact that there was a significant range for the two values for each DRC, we are able to suggest minimum values for the amount of time per day that the plasma concentration exceeds 3 µM as 23 h in each 24 h and, for 10 µM, 9 h in each 24 h.

It might appear logical that a single TDM sample timed to coincide with Tt should be useful as a predictor of PK indices; for example, if the Tt value exceeded a defined threshold, such as an EC value of 1 µM, it could be inferred that adequate plasma concentrations of GS-44 were maintained throughout the inter-dose interval. However, the available data indicate that this assumption is not supported, as no consistent correlation has been observed between Tt values and clinical outcome or disease complexity, albeit based on a limited number of cases [8]. As we have shown that the concentration vs. time graph may vary depending on an individual’s LADME, these observations are not unexpected.

However, using paired TDM samples, other indices become available: AUC0–24 and time over which concentration exceeds certain values (T > 3 µM and T > 10 µM). These are indicators of the physical amount of GS-44 present in the plasma compartment, and by inference, other compartments [8] over the inter-dose period.

For an antiviral drug with similar properties to GS-44 (e.g., ganciclovir), TDM pair sampling is being investigated as a useful tool to ensure that dosages are appropriate for individual patients [41]. Ganciclovir is also absorbed and metabolized into an active drug triphosphate with a long half-life, similar to GS-443902 (the active intracellular GS-44 triphosphate ultimately responsible for viral RNA replication), yet it is administered twice daily in order to ensure that plasma concentrations do not fall to low levels (which encourages viral resistance to develop) or peak at toxic values [41], so it would not be unreasonable to suggest twice daily dosing of GS-44 in FIP treatments.

Practical clinical doses must be determined from PK indices derived from PK studies involving sick animals rather than healthy experimental subjects [8]; hence, the TDM pair sampling method is a valuable tool, both to inform about an individual cat’s dosage and to develop population PK (PopPK) models.

Our results suggest that a target AUC0–24 of 220 µM·h, as indicated by the OPT DRC, might be an appropriate index, with a target of 23 h per 24 h that the plasma GS-44 concentration exceeds 3 µM h, and 9 h per 24 h for exceeding 10 µM.

## 5. Limitations

Although these findings provide valuable preliminary insights into the pharmacokinetic behaviour of GS-44 in cats with FIP, several limitations must be acknowledged when interpreting the data. These constraints arise from both biological variability and practical factors associated with the study design.

A primary limitation stems from the nature of the study population. The cats enrolled were privately owned and, therefore, subject to a range of uncontrolled or unrecorded variables, including differences in environment, diet, and compliance with prescribed dosing schedules. In addition, reported dosing and sampling times were frequently recorded as rounded whole hours (e.g., 07:00, or 13:00), and the actual times of drug administration or sample collection likely varied by several minutes or more.

Additional uncontrolled variables, such as the timing of feeding (and food composition) relative to dosing, concurrent medications, and differences in gastrointestinal health, may also have influenced drug absorption and bioavailability. These factors introduce an unavoidable degree of uncertainty into the pharmacokinetic analyses; however, given the inherent interindividual variability already observed among the study subjects, these sources of error are unlikely to materially alter the overall conclusions.

Another source of limitation lies in the heterogeneity of the study population. The cats varied in age, sex, breed, and clinical presentation, as well as in husbandry and overall health status. This diversity reduced the number of data points available for any single combination of covariates, thereby limiting the statistical power to identify or model specific influences on PK parameters. As data collection continues (the study is ongoing) and the dataset expands, these limitations are expected to diminish. A larger and more homogeneous dataset will support the development of more sophisticated PopPK models, allowing for better identification of covariates affecting GS-44 disposition and facilitating more accurate dose optimization.

Finally, limitations related to the global availability of the analytical method must be considered. The use of HPLC (using either diode array or mass spectroscopy detection) for assaying GS-44 in routine clinical or diagnostic settings will be constrained by cost and equipment availability. Nonetheless, the methodology represents an important step toward establishing accessible, reproducible assays that can support TDM in feline patients. Comparable HPLC-based approaches have been successfully applied by other research groups [4], and wider adoption of such techniques may increase assay availability globally, improving PK understanding and clinical management of FIP.

## 6. Future Prospects

Future work (sister publications in preparation) will focus on expanding the PK dataset to populate a broad range of clinical presentations and treatment regimens, enabling the development of a robust PopPK model for GS-44 in cats with FIP. Such modelling will allow covariate analysis to identify factors influencing drug LADME, such as age, sex, FIP phenotype, route of administration and concurrent disease, facilitating evidence-based dose individualization.

Integration of TDM with clinical outcome data will enable refinement of PK/PD indices, such as AUC0–24/EC100 ratios and time-above-threshold values (T > 3 µM, T > 10 µM), to define PD targets for optimal antiviral efficacy. These refined indices could underpin clinical decision-support algorithms, allowing veterinary practitioners to tailor GS-44 therapy in real time.

Looking ahead, similar TDM-based approaches should be extended to other antiviral compounds being evaluated for FIP treatment, such as EIDD-1931 (which is the active metabolite of molnupiravir). Monitoring plasma concentrations of EIDD-1931 and comparing its exposure–response relationships with those of GS-44 could provide valuable insights into potential combination or sequential treatment regimens, improve understanding of drug–drug interactions, and support early detection of emerging viral resistance.

Importantly, the inclusion of TDM samples from cats treated in different geographic regions would provide a unique opportunity to capture inter-population variability and assess antiviral performance against emerging viral variants, including novel recombinant strains such as FCoV-23. Establishing an international TDM database could therefore enhance global surveillance of pharmacokinetic behaviour, therapeutic response, and resistance development across diverse viral genotypes.

Finally, the accessibility of validated, cost-effective analytical assays remains a key priority. Wider adoption of HPLC-based or simplified point-of-care analytical methods would enhance global implementation of TDM for antivirals used in FIP. Collaborative international efforts to standardize protocols, share pharmacokinetic data, and harmonize analytical validation criteria will be essential to improve therapeutic precision, prevent antiviral resistance, and further reduce FIP-associated mortality.

The principal focus of this study has been the identification of sub-optimal GS-44 exposures (its sister publication into clinical outcomes will determine whether sub-optimal GS-44 exposures compromise outcomes). The converse scenario, supra-optimal plasma concentrations, also warrants consideration. However, GS-44 demonstrates a wide therapeutic margin, with in vitro cytotoxicity reported only at concentrations exceeding 100 µM, and such levels were not observed in this study (the maximum Cmax result was <50 µM). Nonetheless, isolated reports of GS-44 urinary stone formation in vivo [29,30] indicate that excessively high local or systemic concentrations may still present clinical risks, even below cytotoxic thresholds. Supra-optimal dosing also carries financial implications, particularly given the cost of this antiviral therapy, and may limit access to treatment for affected cats. Maintaining GS-44 plasma concentrations within an optimal therapeutic window is therefore essential to balance efficacy, safety, and cost-effectiveness. Continued refinement of dosage regimens through TDM and PopPK modelling will be critical to achieving precision dosing and ensuring sustainable, evidence-based management of FIP.

## 7. Conclusions

Paired TDM samples provide valuable insight into individual variability in GS-44 pharmacokinetics, allowing real-time identification of sub-optimal and supra-optimal exposures. This facilitates dose adjustments tailored to each patient, with the potential to enhance clinical outcomes and, when performed longitudinally, to inform decisions on treatment duration (see its sister publication into clinical outcomes; manuscript under preparation).

The concept of the “optimal cat” (OPT DRC), representing individuals that respond to standard GS-44 dosages and exhibit pharmacokinetic indices consistent with the population mean, accounting for approximately 67% of TDM pairs, is closely aligned with a Gaussian expectation of 68%. However, the substantial variability observed across all dose–response categories underscores the absence of a true “average cat” and reinforces the value of TDM in detecting outliers who might otherwise experience therapeutic failure or unnecessary drug overexposure.

Notably, the proportion of cats classified as LOA N15 DRC (11%) closely mirrors the reported treatment failure rate of 10–15%, suggesting that a proportion of these failures may be attributable to sub-optimal systemic exposure. Early identification of such cases through TDM and subsequent dosage optimization could potentially improve remission rates, leading to near 100% success at treating this disease that was invariably fatal less than five years ago.

Given the very limited repertoire of safe and effective antiviral agents available for veterinary use, the long-term efficacy of GS-44 must be safeguarded. Sub-optimal dosing regimens represent a key risk factor for the emergence of antiviral resistance, an outcome with parallels to antimicrobial resistance in bacteria. Ensuring adequate exposure through evidence-based dosing and widespread implementation of TDM could be essential in preserving the clinical utility of GS-44 and sustaining effective treatment options for FIP worldwide.

## Figures and Tables

**Table 1:** A dose of GS-441524 was recorded as being administered to study cats, as well as a dose per dose (mg/kg/dose) and a dose administered over 24 h (mg/kg/day).

	Dose mg/kg/dose	Dose mg/kg/day
Mean	11.98	21.80
Minimum	2.76	4.80
Maximum	28.00	55.98
Standard deviation	3.95	8.37

**Table 2:** Distribution of GS-441524 (GS-44) sample result cohorts (RCs), both assayed (raw) and normalized to 15 mg/kg (N15), with percentage of total. Samples include those with the GS-44 concentration falling below the lowest range of healthy cat results (LOW raw RC and LOW N15 RC), those within the range of healthy cat results (OPT raw RC and OPT N15 RC), and those above the highest range of healthy cat results (HIGH raw RC and HIGH N15 RC).

	LOW Raw RC	OPT Raw RC	HIGH Raw RC	LOW N15 RC	OPT N15 RC	HIGH N15 RC
Number of values	145	539	44	81	563	84
% of total	20%	74%	6%	11%	77%	12%

**Table 3:** Number and percentage of samples within each dose–response cohort (DRC) as derived from response cohorts (RCs). **Low absorbing (LOA DRC),** LOW raw RC values, which, when normalized to a 15 mg/kg/dose, remained below the lower range of the healthy cat results. **Low dose (LOD DRC),** LOW raw RC values, which, when normalized to a 15 mg/kg/dose, fell within the range of the normal cat results. **High dose (HIGH DRC),** HIGH raw RC values, which, when normalized to a 15 mg/kg/dose, fell within the range of the normal cat results. **High absorbing (HIA DRC),** HIGH raw RC values, which, when normalized to a 15 mg/kg/dose, remained above the upper range of the normal cat results. **Optimal (OPT DRC),** OPT RC values, which remained within the range of the normal cat results after normalizing to a 15 mg/kg/dose.

	LOA DRC	LOD DRC	HIGH DRC	HIA DRC	OPT DRC
Number of values	81	69	41	46	491
% of total	11%	9%	6%	6%	67%

**Table 4:** Calculated maximum concentration (Cmax) (with descriptive statistics) of GS-441524 concentrations attained after each dose event (µM) with both raw and normalized to 15 mg/kg (N15) values for each dose–response cohort (DRC); optimum (OPT Cmax raw/N15), low absorber (LOA Cmax raw/N15), low dose (LOD Cmax raw/N15), high absorber (HIA Cmax raw/N15), and high dose (HIGH Cmax raw/N15).

	Cmax OPT Raw	Cmax LOA Raw	Cmax LOD Raw	Cmax HIA Raw	Cmax HIGH Raw
Mean	15.99	7.64	8.32	20.56	28.46
Minimum	6.74	1.56	4.74	10.19	14.14
Maximum	28.85	15.39	13.84	42.55	43.81
Std. Deviation	5.69	4.01	2.52	9.05	8.31
Std. Error of Mean	0.57	0.77	0.54	2.26	2.51
	**Cmax OPT N15**	**Cmax LOA N15**	**Cmax LOD N15**	**Cmax HIA N15**	**Cmax HIGH N15**
Mean	19.29	8.57	14.98	30.14	27.22
Minimum	8.55	2.33	8.28	18.09	17.16
Maximum	29.46	18.58	24.46	50.54	43.81
Std. Deviation	5.50	4.03	3.72	10.88	8.05
Std. Error of Mean	0.56	0.78	0.79	2.72	2.43

**Table 5:** Calculated half-life (t1/2) hours (h) of GS-441524 elimination for each dose–response cohort (DRC); optimum (OPT t1/2), low absorber (LOA t1/2), low dose (LOD t1/2), high absorber (HIA t1/2), and high dose (HIGH t1/2).

	t1/2 OPT	t1/2 LOA	t1/2 LOD	t1/2 HIA	t1/2 HIGH
Mean	5.87	8.22	4.27	15.44	9.29
Minimum	2.56	1.78	2.63	3.41	1.96
Maximum	36.77	45.19	12.97	82.07	19.45
Std. Deviation	4.05	10.67	2.14	22.61	4.97
Std. Error of Mean	0.41	2.05	0.46	6.82	1.24

**Table 6:** Calculated true trough (Tt) of GS-441524 for steady-state dosing (µM) for each dose–response cohort (DRC); optimum (OPT Tt), low absorber (LOA Tt), low dose (LOD Tt), high absorber (HIA Tt), and high dose (HIGH Tt).

	t1/2 OPT	t1/2 LOA	t1/2 LOD	t1/2 HIA	t1/2 HIGH
Mean	4.02	1.27	1.33	7.43	11.12
Minimum	0.47	0.00	0.07	0.02	4.13
Maximum	12.12	3.44	3.27	19.33	16.07
Std. Deviation	2.09	0.93	0.74	4.06	4.21
Std. Error of Mean	0.21	0.18	0.16	1.01	1.27

**Table 7:** Calculated (AUC0–24) (µM·h) for each dose–response cohort (DRC) and for both raw and N15 concentrations; optimum DRC (OPT AUC raw/N15), low absorber DRC (LOA AUC raw/N15), low dose DRC (LOD AUC raw/N15), high absorber DRC (HIA AUC raw/N15), and high dose DRC (HIGH AUC raw/N15).

RAW	OPT AUC0–24 Raw	LOA AUC0–24 Raw	LOD AUC0–24 Raw	HIA AUC0–24 Raw	HIGH AUC0–24 Raw
Mean	219.00	88.48	98.92	301.60	449.00
Minimum	104.90	22.15	43.92	190.60	276.60
Maximum	435.20	183.60	157.40	578.70	621.60
Std. Deviation	75.38	39.40	27.80	96.39	91.21
Std. Error of Mean	7.61	7.58	5.93	24.10	27.50
**N15**	**OPT** **AUC0–24** **N15**	**LOA** **AUC0–24** **N15**	**LOD** **AUC0–24** **N15**	**HIA** **AUC0–24** **N15**	**HIGH** **AUC0–24** **N15**
Mean	266.00	102.00	178.90	451.80	432.00
Minimum	130.90	32.44	90.98	229.80	272.20
Maximum	438.70	185.40	287.70	821.50	671.90
Std. Deviation	78.50	39.99	47.77	132.60	103.40
Std. Error of Mean	7.93	7.70	10.18	33.16	31.18

**Table 8:** Calculated time over a 24 h period that concentration exceeds 3 µM and 10 µM (h) for each dose–response cohort (DRC); optimum DRC (OPT T > 3 µM/10 µM), low absorber DRC (LOA T 3 µM/10 µM), low dose DRC (LOD T > 3 µM/10 µM), high absorber DRC (HIA T > 3 µM/10 µM), and high dose DRC (HIGH T > 3 µM/10 µM).

3 µM	OPT T > 3 Raw	LOA T > 3 Raw	LOD T > 3 Raw	HIA T > 3 Raw	HIGH T > 3 Raw
Mean	22.63	12.61	14.89	23.27	24.00
Minimum	16.03	0.00	5.25	12.24	24.00
Maximum	24.17	24.00	24.00	24.00	24.00
Std. Deviation	2.29	7.56	4.57	2.94	0.00
Std. Error of Mean	0.23	1.45	0.97	0.74	0.00
**10 µM**	**OPT** **T > 10 Raw**	**LOA** **T > 10 Raw**	**LOD** **T > 10 Raw**	**HIA** **T > 10 Raw**	**HIGH** **T > 10 Raw**
Mean	8.56	0.84	0.53	14.99	21.94
Minimum	0.00	0.00	0.00	0.94	12.43
Maximum	24.00	7.16	2.63	24.00	24.00
Std. Deviation	6.41	1.89	0.87	6.46	3.93
Std. Error of Mean	0.65	0.36	0.18	1.62	1.19

## Data Availability

Data will be made available upon request to SWC@microsampling-laboratory.co.uk.

## Supplementary Materials

The following supporting information can be downloaded at: https://www.mdpi.com/article/10.3390/pathogens15030291/s1, Figure S1: Sample GS-441524 concentration (µM) v time (h) after dose administered, assay values normalized to a 10 mg/kg per dose to remove dosage variability. Faint green and blue dotted lines indicate data obtained from “healthy cats”, extracted by digitization of published data [3,7] and scaled to a 20 mg/kg dose (see Figure 1 and Figure 2). Markers at 1, 3, and 10 µM indicate concentrations [3,7], bracketing effective viral inhibition in vitro values; Figure S2: Sample GS-441524 concentration (µM) v time (h) after dose administered, assay values normalized to a 20 mg/kg per dose to remove dosage variability. Faint green and blue dotted lines indicate data obtained from “healthy cats”, extracted by digitization of published data [3,7] and scaled to a 20 mg/kg dose (see Figure 1 and Figure 2). Markers at 1, 3, and 10 µM indicate concentrations [3,7], bracketing effective viral inhibition in vitro values. Table S1: Volume of distribution (Vd) of GS-441524 by dose–response cohort (DRC); Table S2: Clearance at steady state (Clss) of GS-441524 by dose–response cohort (DRC). Table S3: Elimination rate constant (Kel) of GS-441524 by dose–response cohort (DRC).
