# Supplementary material for: Therapeutic Drug Monitoring of GS-441524 in Cats with Feline Infectious Peritonitis: Pharmacokinetic Variability and Implications for Dose Optimization
Source: Pathogens. 2026 Mar 6;15(3):291. doi: 10.3390/pathogens15030291 (PMC13028839; doi:10.3390/pathogens15030291)
Supplement: Supplementary file 1 [file pathogens-15-00291-s001.zip › pathogens-4112827-supplementary.pdf]

Supplementary material:

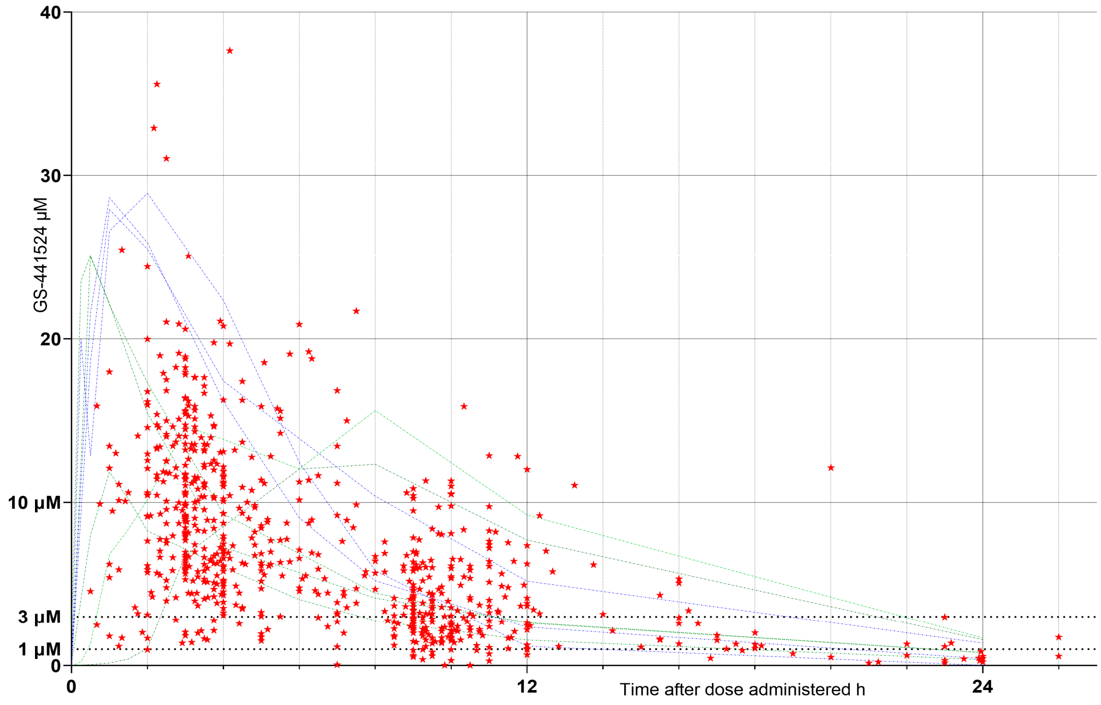

Figure S1 Sample GS-441524 concentration (µM) v time (h) after dose administered, assay values normalised to 10 mg/kg per dose to remove dosage variability. Faint green & blue dotted lines indicate data obtained from 'healthy cats', extracted by digitization of published data [3,7] and scaled to a 20 mg/kg dose (see Figures 1 and 2). Markers at 1, 3 & 10 µM indicate concentrations [3,7] bracketing effective viral inhibition in vitro values.

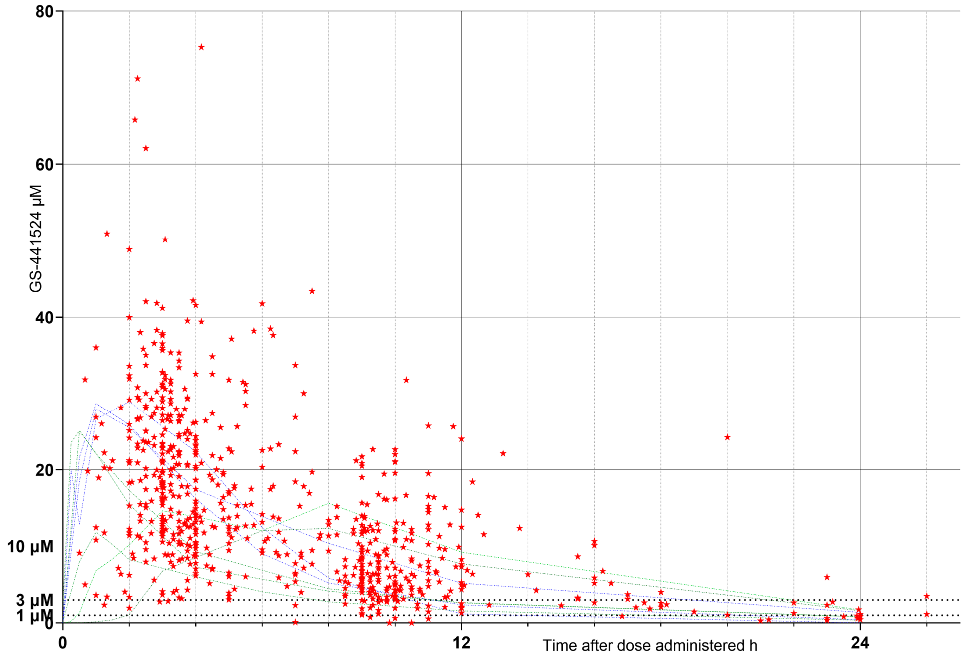

Figure S2 Sample GS-441524 concentration (µM) v time (h) after dose administered, assay values normalised to 20 mg/kg per dose to remove dosage variability. Faint green & blue dotted lines indicate data obtained from 'healthy cats', extracted by digitization of published data [3,7] and scaled to a 20 mg/kg dose (see Figures 1 and 2). Markers at 1, 3 & 10 µM indicate concentrations [3,7] bracketing effective viral inhibition in vitro values.

Table S1 Volume of distribution (Vd) of GS-441524 by dose response cohorts (DRC)

	OPT Vd	LOA Vd	LOD Vd	HIA Vd	HIGH Vd
Mean	3.58	12.26	3.75	3.21	5.54
Minimum	1.51	2.10	1.79	0.73	1.06
Maximum	24.69	54.64	7.70	6.89	30.02
Number of values	98	27	22	16	11

Table S2 Clearance at steady state (Clss) of GS-441524 by dose response cohorts (DRC)

	OPT Clss	LOA Clss	LOD Clss	HIA Clss	HIGH Clss
Mean	0.43	1.12	0.62	0.24	0.25
Minimum	0.22	0.48	0.41	0.13	0.15
Maximum	0.80	3.20	0.90	0.40	0.35
Number of values	98	27	22	16	11

Table S3 Elimination rate constant (Kel) of GS-441524 by dose response cohorts (DRC)

	OPT kel	LOA kel	LOD kel	HIA kel	HIGH kel
Mean	0.14	0.17	0.18	0.11	0.09
Minimum	0.02	0.02	0.05	0.04	0.01
Maximum	0.27	0.39	0.26	0.35	0.20
Number of values	98	27	22	16	11
